# Development and validity evidence of the multidimensional scale of sexual self-concept in a Spanish-speaking context

**DOI:** 10.1186/s41155-019-0136-1

**Published:** 2019-12-05

**Authors:** Rodrigo Ferrer-Urbina, Geraldy Lorena Sepúlveda-Páez, Diego-Tomás Henríquez, Daniel Ignacio Acevedo-Castillo, Débora Alejandra Llewellyn-Alvarado

**Affiliations:** 1 Escuela de Psicología y Filosofía, Universidad de Tarapacá, 18 de septiembre #2222, Arica, Chile; 2 Carrera de Psicología, Universidad de Tarapacá, Arica, Chile

**Keywords:** Sexual self-concept, Sexual self-esteem, Sexual self-efficacy, Sexual assertiveness, ESEM

## Abstract

**Background/objective:**

STIs and HIV/AIDS are an important public health problem, transmitted by risky sex behaviours. In this context, it is necessary to identify protective factors, of those behaviours, as sexual self-concept. Sexual self-concept is a multidimensional trait (i.e. sexual self-esteem; sexual self-efficacy; and sexual assertiveness), but, in an extensive review, we did not find any measure to assess this multidimensional construct in a Spanish-speaking context. The objective of this research is development a scale to assess sexual self-concept in young people and adults.

**Method:**

Time-space sampling with a total size of 792 participants, coming from the two Chilean cities (i.e. Arica and Iquique) with the highest HIV rates, aged between 17 and 53 years old (ME = 23.42; SD = 6.33), with 66.2% women (*N* = 500), 33.6% men (*N* = 258).

**Results:**

Final scale has 16 items and 4 dimensions: sexual self-esteem, sexual self-efficacy, assertive sexual communication, and assertive sexual behaviour. The identified structure provides satisfactory levels of reliability (ω > .8) and presents robust evidence of validity, based on the internal structure of the test, using ESEM (RMSEA = .060; CFI = .99; TLI = .98), evidence of validity based on relationship to other variables (i.e. risky sexual behaviour) and measurement invariance between men and women.

**Conclusions:**

The multidimensional scale of sexual self-concept has adequate psychometric properties to assess sexual self-concept in equivalent samples.

## Introduction

Sexually transmitted infections (STIs), including human immunodeficiency virus (HIV) and its active manifestation, acquired immunodeficiency syndrome (AIDS), are health problems to millions of people (Lee et al., 2017). Despite public and private efforts to control STIs and HIV/AIDS, available evidence indicates that both problems are still growing (Del Romero et al., 2019).

In Chile, STIs, including HIV/AIDS, are concentrated mainly in urban districts of the country (Ministerio de salud, 2010), with a heterogeneous regional distribution, with the highest rates of HIV/AIDS in the first two regions of the north end, being the rates of new notifications of HIV/AIDS, per 100,000 inhabitants, of 54.4 in Arica and Parinacota and 48.3 in Tarapacá regions, while was 27.7 in the whole of Chile (ISP, 2016).

STIs and HIV/AIDS are concentrated in young people more than any other sexually active age group, observing the highest prevalence in those aged 20–29 years (Elattabi et al., 2017), which is associated with being the group with the highest frequency of risky sex behaviour (Folch et al., 2015); this is why they are the group of greatest interest for public health policies on STIs and HIV/AIDS (Ministerio de salud, 2018).

STIs and HIV can be contracted in multiple ways, although the main route of transmission is through direct sexual contact with carriers without the proper use of protective barriers, that is, risky sexual behaviours (Folch et al., 2015). In this context, behavioural sciences have made efforts to identify and modify psychological factors associated with sexual risk behaviours, developing multiple research focused on identifying risk factors, such as poor sex education and early sexual initiation (e.g. Shegog et al., 2017). However, there is broad evidence that prevention should focus not only on reducing risk factors but also on promoting protective factors, such as the organisation of gender identities and roles (e.g. Alimoradi et al., 2017; Rohleder et al., 2017), parental communication (e.g. Simons et al., 2016), access to information (e.g. Villegas et al., 2016), and active participation in organisations (e.g. Frumence et al., 2014).

Within these protective factors, one that has been consistently referred is sexual self-concept (e.g. Hensel et al., 2011; O’Sullivan et al., 2006). Self-concept can be considered as a multidimensional construction that refers to the perceptions and positive/negative feelings that an individual has as a sexual being (e.g. Talley and Stevens, 2017), bringing together a large number of concepts (e.g. sexual-anxiety, sexual self-efficacy, sexual self-consciousness, sexual-preoccupation, sexual self-assertiveness, sexual self-esteem), ranging from proposals that include a few dimensions (e.g. Ziaei et al., 2013; Rostosky et al., 2008) to ones that include as many as 20 (e.g. Snell, 1998).

Among the multiple aspects included in the definitions of sexual self-concept, those that present the most studies in their relationship with risky sexual behaviours and STI studies are those that allude to the following: feelings about one’s own sexuality (i.e. sexual self-esteem) (e.g. Bermudez et al., 2019; Rohleder et al., 2017); beliefs about one’s own sexual ability (i.e. sexual self-efficacy) (e.g. Espinosa et al., 2019; Rosenthal et al., 2012; Træen et al., 2014); and expressions of own sexual desires (i.e. sexual assertiveness) (e.g. Uribe-Alvarado et al., 2017; Santos-Iglesias and Sierra, 2010).

In general terms, self-esteem refers to a subjective and individual experience corresponding to the evaluation that each person makes of themselves, which can be positive or negative, constructed from the interaction of the subject with their social and cultural environment (Donnellan et al., 2011). Sexual self-esteem refers to the positive evaluation of oneself that gives the ability to experience sexuality in a healthy and satisfying way (e.g. Brassard et al., 2015). Sexual self-esteem has been associated with lower risk sexual behaviour, favouring the use of contraceptives (Adler and Hendrick, 1991); it seems that those who accept themselves as sexually active can prepare for sexual interactions in a favourable and safe way (Toro et al., 2008).

Self-efficacy is a concept developed by Bandura and National Inst of Mental Health (1986) in his cognitive social theory and refers to perceptions of one’s own skills and abilities (Schwarzer, 1992). In sexual domain, self-efficacy refers to belief in one’s capacity to successfully achieve behaviours and affective responses in a sexual context (Bailes et al., 2011). The concept of sexual self-efficacy is closely related to safe sexual practices (Soler et al., 2000). In addition, the high levels of sexual self-efficacy are related to a lower probability of developing risky sexual behaviour (Rosenthal et al., 2012).

In the case of sexual assertiveness, there are two different facets: (1) assertive sexual behaviour, which refers to the ability to initiate sexual activity, reject unwanted sexual activity, and negotiate the desired sexual behaviour (Morokoff et al., 1997); and (2) assertive sexual communication, which refers to the social skills that facilitate the communication of preferences or needs between sexual partners (Loshek and Terrell, 2015; Santos-Iglesias et al., 2014). Both aspects have been shown to predict the use of condoms, safe sexual practices (Kelly and Kalichman, 1995), lower risk sexual behaviour, HIV (Brown et al., 2018), and greater sexual satisfaction (Sánchez-Fuentes et al., 2016).

In order to identify and study these factors, it is necessary to have measurement instruments to evaluate them at low cost and based on evidence. The latter implies carrying out the necessary procedures to ensure that the inferences and interpretations of the observed scores are adequate and have the minimum ethical guarantees to support the conclusions and decisions that derive from the measurement process (American Educational Research Association, American Psychological Association,, and National Council on Measurement in Education, 2014). These guarantees correspond to two essential aspects: reliability and evidence of validity (Prieto and Delgado, 2010).

Currently, in the world-wide literature, there are multiple measurement instruments designed to evaluate sexual self-concept (e.g. Snell, 1998; Hensel et al., 2011; Ziaei et al., 2013) and others focused on specific factors, mainly: sexual self-esteem (e.g. Bornefeld-Ettmann et al., 2018); sexual self-efficacy (e.g. Bailes et al., 2011); and sexual assertiveness (e.g. Hurlbert, 1991; Morokoff et al., 1997; Quina et al., 2000; Santos-Iglesias et al., 2014). However, none of them is adapted, with evidence of reliability and validity, for use in the Spanish-speaking population.

In this context, and given that the development of measuring instruments in a particular culture may present advantages over linguistic adjustments (e.g. Cohen et al., 2007), the purpose of this work is to develop a multidimensional scale for the assessment of sexual self-concept, based on the most researched dimensions of sexual self-concept, with evidence of validity based on internal structure and relationship to other measures, for use in Spanish-speaking young people and young adults, in order to promote the development of research and intervention related to the prevention of STIs and HIV/AIDS in at-risk populations.

## Method

### Design and participants

This study has a cross-sectional and instrumental design (Ato et al., 2013).

A time-space sample, also referred to as time-location sample (Semaan, 2010), was used. Seven hundred ninety-two young people and adults from the largest cities in the far north of Chile: Arica (*n* = 424) and Iquique (*n* = 336), regional capitals of Arica and Parinacota and Tarapacá, respectively. 90.1% (*n* = 718) claimed be heterosexual, 87.7% (*n* = 666) claimed to have had sex in the last 2 years, and 59.8% (*n* = 484) claimed to currently have at least one sexual partner. In Arica, 301 (71%) participants were women, 121 (28.5%) men, and 3 (0.5%) intersexuals, with a mean age of 25.10 years (SD = 7.43); in Iquique, 199 (59.2%) were women and 137 (40.8%) men, with a mean age of 21.62 years (SD = 4.08).

### Instruments

*Multidimensional scale of sexual self-concept*: ad-hoc developed scale, designed to assess 4 dimensions: (1) sexual self-esteem; (2) sexual self-efficacy; (3) assertive sexual behaviour; (4) assertive sexual communication. Reagents are 4-point Likert behavioural/attitudinal statements (1 = ‘Never’ to 4 = ‘Always’; 1 = ‘Strongly disagree’ to 4 = ‘Strongly agree’).

Initially, 74 items were created (20 items for sexual self-esteem; 19 for sexual self-efficacy; 15 for assertive sexual behaviour; and 20 for assertive sexual communication), which were evaluated by four judges with experience in psychometrics, who suggested keeping 53 items, with which a pilot application was developed online, in university students samples (*n* = 210). Then, the scale was refined, based on items analysis and reliability. Finally, a 38-item version was applied for this study (see Additional file 1). The final version and its psychometric evidences are reported in the ‘Results’ section.

*Sexual risk behaviour scale* (Ferrer-Urbina et al., 2019): a 12-item scale, designed to assess three dimensions of risky sexual behaviour: (1) sexual activity with multiple partners (items = 4); (2) inadequate or insufficient use of protection barriers (items = 4); and (3) sexual activity under the influence of alcohol or drugs (items = 4). The questions are behavioural/attitudinal statements on a Likert-type scale of 4 points (0 ‘never’ to 3 ‘always’), conditioning to report only the behaviour of the past 2 years. Scale reported validity evidence based on the internal structure and good reliability (ω > .8) (Ferrer-Urbina et al., 2019).

### Procedure

Eight 4th- and 5th-year psychology students were trained to provide instructions, answer participants’ questions, and apply both pencil and paper questionnaires, in Arica and Iquique cities. Participants were contacted by surveyors in the recreation areas of higher education institutions, explaining the objectives of the study and inviting them to respond on the spot. Informed consent was applied, where research objectives, participant´s rights, confidentiality, and anonymity were stated. Anonymity was safeguarded by anonymous return in a sealed envelope, without any form of personal identification. The response procedure lasted less than 15 min.

Both the instrument and the entire procedure were known and approved by the scientific ethics committee of the University of Tarapacá.

### Statistical analysis

To test the internal structure of the scale, an exploratory structural equation modelling (ESEM) with TARGET rotation (Asparouhov and Muthén, 2009) and robust weighted least squares estimation method (WLSMV), which is robust with non-normal discrete variables (Asparouhov, 2007), was made from the polychoric correlation matrix (Barendse, Oort, and Timmerman, 2015). In addition, to evaluate the plausibility of the integration of scale dimensions, a second-order confirmatory factor analysis (CFA) was performed, using WLSMV estimation method and the polychoric correlation matrix. Overall model fit was assessed using the following indices: comparative fit index (CFI), Tucker-Lewis index (TLI), and root mean square error of approximation (RMSEA), cut-off point recommendations of Schreiber (2017) (e.g. CFI > .95; TLI > .95; RMSEA<.06). Reliability was estimated, for each dimension, through Cronbach’s alpha and McDonald’s hierarchical omega coefficients, in their non-ordinal versions. In order to assess the stability of the scale between people of different sexes, scale and metric invariance testing were performed, regarding decreases in CFI under .005 and increases in RMSEA under .010 as evidence of invariance (Chen, 2007). Finally, evidence of validity based on convergence was established from a structural equation model of the relationship of scale dimensions with dimensions of the sexual risk behaviour scale, using WLSMV estimation method and the polychoric correlation matrix. All the analyses were carried out using the MPlus program (7.4).

## Results

Table 1 shows the settings of the ESEM measurement models, both in the original version (38 items) and in the debugged version (16 items). Debugged scale was tested, in two models: covariate ESEM and second-order CFA.

**Table 1:** Global fit of measurements models

Model	*N* Par	χ^2^	DF	*P*	CFI	TLI	RMSEA	RMSEA CI 90%	
								Low	Up
ESEM (38)	262	2845.187	557	.00	.96	.95	.072	.069	.075
ESEM (16)	107	236.530	62	.00	.99	.98	.060	.052	.068
Second-order CFA (16)	67	2030.898	101	.00	.91	.89	.155	.149	.161

*N* Par, numbers of parameters in the model; *χ*^2^ = chi-squared; degrees of freedom; *P*, chi-squared NHT probability; *CFI*, comparative fit index; *TLI*, Tucker-Lewis index; *RMSEA*, root mean square error of approximation

According to the most common fit criteria in the literature (CFI > .95; TLI > .95; RMSEA < .06: Schreiber et al., 2006), the original model is not an enough explanation of the observed covariation matrix; therefore, debugging of the instrument was carried out by considering three criteria: (1) selection of strong factor loadings (*λ* > .5); (2) removal of redundant items; and (3) removal of items whose cross-loadings were strong (> .3).

The final debugged scale consisted of 16 items divided into four dimensions (sexual self-esteem, sexual self-efficacy, assertive sexual behaviour, and assertive sexual communication), each comprising 4 items. Fit indicators (Table 1), both comparative (CFI; TLI) and absolute (e.g. χ^2^/DF; RMSEA), indicate that the debugged ESEM model is a good population representation of the observed relationships, but only in the covariate ESEM version. Factorial loadings, factorial covariances, and reliability estimates for each dimension are presented in Table 2.

**Table 2:** Standardised factorial loadings reliability coefficients (Cronbach’s Alpha and McDonald’s omega) for each dimension

Original item in Spanish for Chilean sample (untested translation for understanding purposes only)	SES	SEF	ASB	ACS
Sexual self-esteem (SSE)				
No cambiaría nada de mi vida sexual actual. (I would not change anything in my current sex life.)	.926**	.052	.006	.345**
Estoy muy satisfecho(a) con mi vida sexual. (I am very satisfied with my sex life.)	.974**	.051	− .093*	− .098*
Estoy bien conmigo mismo(a) en el ámbito sexual. (I’m fine with myself in the sexual domain.)	.673**	− .006	.038*	.030
Mis encuentros sexuales son satisfactorios para mí y mi(s) pareja(s). (My sexual encounters are satisfying for me and my partner(s).)	.556**	.147**	.172**	− .090**
Sexual self-efficacy (SSF)				
Creo que sé estimular bien a mi(s) parejas. (I think I know how to stimulate my partner(s) well.)	− .106*	.701**	− .039	− .035
Me desenvuelvo de buena forma en el ámbito sexual. (I perform well in the sexual realm.)	− .035	.887**	− .013*	− .052
Creo que tengo un buen número de cualidades en el ámbito sexual. (I think I have a good number of qualities in the sexual field.)	.288**	.869**	.118*	− .020
Creo en mis capacidades y habilidades sexuales. (I believe in my abilities and sexual skills.)	.235**	.763**	− .068	.159**
Assertive sexual behaviour (ASB)				
Comienzo la intimidad con mi compañero(a) sexual solo cuando quiero. (I start intimacy with my sexual partner only when I want.)	.097	.047	.724**	.061
Tengo sexo solo cuando quiero, incluso si mi(s) pareja(s) insiste(n) en tenerlo. (I have sex just when I want, even if my partner(s) insists on having it.)	.024	− .051	.977**	− .026
Solo realizo prácticas sexuales que deseo. (I only perform sexual practices that I desire.)	.066	.071*	.655**	− .019
Me mantengo firme a las presiones de mi(s) pareja(s) si no quiero tener relaciones sexuales o algún tipo de intimidad. (I stand firm to the pressures of my partner(s) if I don’t want to have sex or any kind of intimacy.)	− .036*	.016	.685**	.148**
Assertive sexual communication (ASC)				
Manifiesto cuales son mis sentimientos, afectos y deseos sexuales. (I manifest my feelings, affections and sexual desires.)	.026	.093	.137*	.550**
Le(s) digo a mi(s) pareja(s) donde quiero que me toque(n) cuando tenemos sexo. (I tell my partner(s) where I want to be touched when we have sex.)	− .008	.035	− .109*	.922**
Le expreso a mi(s) pareja(s) sexual cuando deseo que me acaricie(n). (I express my sexual partner(s) when I want to be caressed.)	− .063	− .006	− .139*	.865**
Pido lo que quiero durante una relación sexual. (I ask what I want during a sexual relationship.)	.232	− .027	− .065*	.681*
Factorial covariations				
Sexual self-esteem		.758**	.417**	.491**
Sexual self-efficacy			.458**	.608**
Assertive sexual behaviour				.679**
Reliability estimators				
Alpha (*α*)	.903	.897	.808	.844
Omega (*ω*)	.903	.898	.810	.845

**p < .01; *p < .05

The factorial loadings evidence adequate representations of each factor (*λ* > .5), with low levels of cross-loadings (*λ* ≤ .3). Structural relationships are moderate (*r* > .3) or high (*r* > .5) (Cohen, 1988). Reliability estimates are higher than .80 (i.e. ω = .90, sexual self-esteem; ω = .89, sexual self-efficacy; ω = .81, assertive sexual behaviour; ω = .84, assertive sexual communication), showing high levels of internal consistency in all dimensions (Prieto and Delgado, 2010).

Results of measurement invariance testing, between men and women, of the final version of the scale (version of 16 items), are shown in Table 3. CFI and RMSEA deltas non-evidence practical fit changes in metric or scalar model, compared with configural model, so it can be assumed that measurement equivalence between sexes.

**Table 3:** Measurement invariance testing

	*N* Par	χ^2^	DF	*P*	CFI	RMSEA	Δ_χ2_	Δ_DF_	*P*_Δχ2_	Δ_CFI_	Δ_RMSEA_
Configural	140	850.599	196	.000	.971	.092					
Metric	128	851.804	208	.000	.972	.089	10.750	12	.550	.001	− .003
Scalar	100	897.076	236	.000	.971	.084	78.258	40	.000	.000	− .008

*Δ*_*χ2*_, chi-squared delta, from reference model compared with configural (unrestricted) model; *Δ*_*DF*_, degrees of freedom delta, from reference model compared with configural (unrestricted) model; *P*_*Δχ2*_, probability of fit differences, from reference model compared with configural (unrestricted) model; *Δ*_*CFI*_, CFI delta, from reference model compared with configural (unrestricted) model; *Δ*_*RMSEA*_, RMSEA delta, from reference model compared with configural (unrestricted) model

Finally, Table 4 shows the relationships between latent dimensions of the multidimensional scale of sexual self-concept and sexual risk behaviour scale. Proposed model has adequate fit (*χ*^2^_DF = 329_; *p*_χ2_ = .00; CFI = .98; TLI = .97; RMSEA = .05).

**Table 4:** Standardised relationships between latent dimensions of the multidimensional scale of sexual self-concept and sexual risk behaviour scale

	SES	SEF	ASB	ACS
Sexual activity with multiple partners	− .694**	.814**	− .287**	.348**
Inadequate or insufficient use of protection barriers	− .321**	.457**	− .218**	.569**
Sexual activity under the influence of alcohol or drugs	− .535**	.576**	− .177*	.251**

**p < .01; *p < .05

According to the observed relationships, SES has medium (> .3; Cohen, 1988) and large (> .5; Cohen, 1988) inverse effects on risky sexual behaviours; SEF also has medium (< − .3; Cohen, 1988) and large (< − .5; Cohen, 1988) but inverse effects; ASB has only small inverse effects (<− .1; Cohen, 1988); and ACS has small to large direct effects.

## Discussion

The purpose of this study was to develop a multidimensional scale for the assessment of sexual self-concept, for use in Spanish-speaking young people and young adults. Global model’s fit, factor loadings sizes, non-existence of relevant cross-loadings allow supporting the model’s structure, providing evidence, based on the internal structure, for the dimensional scale scores interpretation. In this same direction, dimensional reliability estimates allow us to assume that each dimension has levels of internal consistency that minimise measurement errors, despite being of a reduced length.

Regarding the evidence of validity based on the relationship with other variables, relationships are found according to the literature in the case of the dimensions of sexual self-esteem (e.g. Adler and Hendrick, 1991; Brassard et al., 2015; Toro et al., 2008) and assertive sexual communication (e.g. Loshek and Terrell, 2015). However, in the case of sexual self-efficacy and behavioural assertiveness, inverse relationships are observed, contrary to what is expected in the reference literature. These contradictory results can be attributed to the fact that many of the scales of sexual self-efficacy and behavioural assertiveness are oriented towards the use of condoms (e.g. Baele et al., 2001; Uribe-Alvarado et al., 2017), while, in this scale, the definitions are oriented towards one’s own perceived sexual competence, being probable that those who experience greater sexual activity with multiple partners and, therefore, more risks, possess a perception of greater ability, which would increase their perception of self-efficacy and a more assertive sexual behaviour.

Factorial covariations allow four dimensions of the instrument (sexual self-esteem, sexual self-efficacy, assertive sexual communication, and assertive sexual behaviour) to be considered as interdependent aspects of sexual self-concept, but they must be analysed independently, because the second-order model is an inadequate explanation of the observed relationships, which is reflected in poor adjustment indicators.

According with Chen (2007) invariance suggested standards, structural and metric measurement invariance, between sexes, are supported, making possible to apply the scale to both men and women, since factorial loadings are equivalent between groups.

The main restriction of this study corresponds to the non-probabilistic sampling used, which makes it necessary to realise new psychometric studies in order to increase the generalisation of the scale. It is therefore recommended that this instrument be applied, when appropriate, in health, medical, and educational contexts in order to obtain additional evidence of validity and representativeness in new contexts (e.g. new countries, high-risk populations, other languages, migrants).

Finally, despite contextual limitations, this scale is not only a new instrument developed according to the current state of the psychometric techniques, but it also establishes a novel proposal that collects and integrates a broad spectrum of specific sexual health concepts that provide good evidence for their potential role in risky sexual behaviour, with the notorious advantage of being a short scale, which can be easily incorporated into series of measuring instruments, in health contexts, where quick applications are required in conjunction with other scales.

### Conclusions

The final version (16 items) of the multidimensional scale of sexual self-concept has evidence of reliability, validity, based on the internal structure of the test and on the convergence with other measures, and measurement invariance between sexes, which support the interpretation of scores in equivalent samples of women and men. Initial evidence suggests that the current scale can be used for the development of research on psychological factors involved in sexual behaviours.

## Supplementary information

**Additional file 1.** Original Spanish scale 38.

## Data Availability

The datasets used and analysed during the current study are available to general use.
