# Supplementary material for: Development and validity evidence of the multidimensional scale of sexual self-concept in a Spanish-speaking context
Source: Psicol Reflex Crit. 2019 Dec 5;32:22. doi: 10.1186/s41155-019-0136-1 (PMC6966990; doi:10.1186/s41155-019-0136-1)
Supplement: Supplementary file 1 — Additional file 1. Original Spanish scale 38. [file 41155_2019_136_MOESM1_ESM.docx]

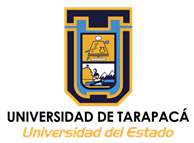
**CONSENTIMIENTO INFORMADO**

**Proyecto FONDECYT nº 11170395: DESARROLLO DE UN MODELO PREDICTIVO DE CONDUCTAS SEXUALES DE RIESGO DE ITS Y VIH/SIDA, A PARTIR DE FACTORES PSICOLÓGICOS RELATIVOS AL ÁMBITO SEXUAL, PARA JÓVENES Y ADULTOS JÓVENES CON RESIDENCIA URBANA EN EL NORTE GRANDE DE CHILE.**

**NOMBRE INVESTIGADOR RESPONSABLE:** Dr. Rodrigo Ferrer Urbina

**UNIDAD RESPONSABLE:** Escuela de Psicología y Filosofía

**INSTITUCIÓN:** Universidad de Tarapacá, Arica

**TELÉFONOS:** +5658- 2205640

**PROPÓSITO DEL ESTUDIO**: El objetivo de esta investigación es identificar factores psicológicos relativos al ámbito sexual, asociados con conductas sexuales de riesgo de contagio de ITS y VIH/SIDA, en jóvenes y adultos jóvenes con residencia urbana en el Norte Grande de Chile.

A) **DESCRIPCIÓN DEL ESTUDIO Y DE SU PARTICIPACIÓN:** Si usted es mayor de edad en Chile (18 o más años) y decide participar del estudio, se le pedirá que firme este consentimiento en dos ejemplares: uno que quedará en su poder y otro para el investigador. Su participación consistirá en responder un conjunto de cuestionarios con preguntas acerca de su opinión y acerca de diferentes preguntas del ámbito de la sexualidad, así como de su propia conducta sexual. Para participar, es fundamental que tenga 18 o más años y que ya haya tenido experiencias sexuales voluntarias. El llenar estos instrumentos le tomará un tiempo aproximado de 25 minutos.

B) **POSIBLES RIESGOS**: Los riesgos de participar en este estudio no son mayores que los que podría encontrarse en la vida cotidiana. Sin embargo, un eventual riesgo podría ser que se sintiera incómodo (a) mientras responda, dado que se le harán preguntas sobre su vida, incluyendo descripciones explícitas sobre su vida sexual. No obstante, usted es libre de dejar el estudio en cualquier momento, sin necesidad de dar ningún tipo de explicación. Cualquier otro efecto que Ud. considere que puede derivarse del uso de las encuestas deberá comunicarlo al Dr. Rodrigo Ferrer Urbina en el teléfono +5658- 2205640.

C) **BENEFICIOS**: La información que usted aporte será de gran valor para ayudar a comprender las conductas sexuales de las personas residentes en el Norte Grande de Chile, así como las opiniones y percepciones asociadas. Esta información nos ayudará a comprender el cómo nos comportamos en la intimidad, de modo de poder orientar realistamente acciones de salud, prevención y mejorar el bienestar sexual de la población de la macroregión.

D) **CONFIDENCIALIDAD Y RESGUARDO DE LA INFORMACIÓN**: Toda la información derivada de su participación será manejada con **estricta confidencialidad** y cada cuestionario sólo se identificará mediante un número con el objeto de salvaguardar el anonimato de quien responde. Sólo los investigadores tendrán acceso a los datos por usted proporcionados. La información será resguardada según todos los requerimientos que la ley explicita y serán depositados por un periodo de 5 años en el repositorio de la Escuela de Psicología y Filosofía de la Universidad de Tarapacá, Sede Arica.

Asimismo, tanto en el análisis como en la publicación y difusión científica de los resultados, no se identificará la identidad de ninguno de los participantes, para así resguardar el **anonimato**.

La información que entregue mediante su participación sólo será utilizada con fines científicos y relativos a esta

Investigación y no será usada con fines ajenos a los explícitamente expresados en este documento. De necesitarse los datos para otros fines se solicitará nuevamente consentimiento informado, y en caso de no ser esto posible, se solicitará autorización al Comité de Ética Científico de la Universidad de Tarapacá.

E) **VOLUNTARIEDAD**: La participación en esta investigación es absolutamente voluntaria y usted puede retirarse en

cualquier momento que lo desee del estudio, sin que ello tenga ninguna consecuencia para Ud.

F) **DERECHOS DEL PARTICIPANTE**: Si usted requiere cualquier otra información sobre su participación en este estudio, si tiene alguna duda al respecto o desea conocer los resultados del mismo, puede contactar al investigador responsable o al Presidente del Comité de Ética Científico de la Universidad de Tarapacá:

Dr. Rodrigo Ferrer U. Dr. Omar Espinoza N.

[rferrer@uta.cl](mailto:rferrer@uta.cl%20) o al teléfono: +5658-2205640. [cec.uta@uta.cl](mailto:cec.uta@uta.cl) o al teléfono: 58-2 386021

Académico de la Escuela de Psicología y Filosofía Presidente Comité Ética Científico

Universidad de Tarapacá Universidad de Tarapacá

Después de haber recibido y comprendido la información de este documento y de haber podido aclarar todas mis dudas, otorgo el consentimiento para participar en el proyecto: “DESARROLLO DE UN MODELO PREDICTIVO DE CONDUCTAS SEXUALES DE RIESGO DE ITS Y VIH/SIDA, A PARTIR DE FACTORES PSICOLÓGICOS RELATIVOS AL ÁMBITO SEXUAL, PARA JÓVENES Y ADULTOS JÓVENES CON RESIDENCIA URBANA EN EL NORTE GRANDE DE CHILE”

Declaro recibir una copia de este documento. Mi consentimiento está dado voluntariamente y no ha sido forzado ni obligado.

Nombre:

Firma: _____

Fecha: _____

______________________________

Firma Investigador Responsable

Dr. Rodrigo Ferrer U.

FONDECYT 11170395

**Estimada(o):**

Esta encuesta **ANÓNIMA** tiene por propósito ayudarnos a conocer algunos aspectos de la vida sexual de las personas. Para cumplir con este propósito, te pedimos que por favor respondas una serie de preguntas, lo que te tomará aproximadamente menos de 10 minutos.

Para que los resultados de esta encuesta sean útiles, es fundamental que respondas con total sinceridad, teniendo la certeza que **NADIE PODRÁ IDENTIFICARTE** (no se pregunta por ningún dato de identificación) y **NADIE TE JUZGARÁ** por tus respuestas.

Muchas gracias por tu esencial participación.

A continuación, aparecen una serie casillas las cuales debe completar y/o marcar con una X de acuerdo a la opción que más te representa.

| **Sexo Biológico** | Mujer | Hombre | | Intersexual |  |
| --- | --- | --- | --- | --- | --- |
| **Edad** |  |  |  |  |  |
| **Ciudad de Residencia** |  |  |  |  |  |
| Arica | Iquique | Otra |  |  |  |
| **Identidad étnica** |  |  | |  |  |
| Aimara | Atacameña | Mapuche | | Quechua | Otra |
| **Estado relacional** |  |  | |  |  |
| Soltero/a | Casado/a | Separado/a | | Conviviente | Otra |
| **Orientación Sexual** |  |  | |  |  |
| Heterosexual | Homosexual | Bisexual | | Transexual | Otra |
| **Actualmente, ¿cuenta con por lo menos una pareja sexual?** | | | | NO | SI |
| **¿Ha tenido sexo en los últimos dos años?** | | | | NO | SI |
| **¿Ha sido diagnosticado con alguna enfermedad de transmisión sexual?** | | | | |  |
| NO | VIH/SIDA | Herpes genital | | Sífilis |  |
| Virus del Papiloma Humano | Gonorrea | Tricomoniasis | | Otra |  |

Antes de responder a las siguientes afirmaciones, es necesario especificar que entenderá por:

**Sexo, acto sexual o práctica sexual:** cualquier actividad en la que ha existido algún tipo de contacto genital y/o anal, en cualquiera de sus formas (penetración, sexo oral, roces entre genitales), incluyendo todo tipo de encuentros sexuales con una o más personas. Ya sea, con una pareja estable, encuentro casual, “amigos(as)”, etc.

**Preservativo:** incluye el condón (preservativo masculino) como el dique de goma (preservativo femenino).

**Penetración:** ésta se refiere a la anal o vaginal, llevada a cabo por el aparato reproductor masculino o cualquier otro objeto que se utilice para ejercer ésta (por ejemplo, juguetes sexuales).

Las preguntas son genéricas, por lo que deben entenderse en un sentido amplio, **SI ALGUNA PREGUNTA INCLUYE A LA PAREJA Y USTED NO TIENE ACTUALMENTE PAREJA, PIENSE EN EXPERIENCIAS** **ANTERIORES PARA RESPONDER**. Además, cabe destacar que, las afirmaciones que se presentan en el estudio están orientadas hacia **RELACIONES SEXUALES CONSENSUADAS.**

SI TIENES DUDAS, SIEMPRE PUEDES VOLVER A MIRAR ESTAS INDICACIONES.

| **Indique con una X, por favor, con qué frecuencia usted realiza las siguientes conductas:** | | | | | |
| --- | --- | --- | --- | --- | --- |
| **N.º** | **ÍTEMS** | **Nunca** | **Alguna Vez** | **Varias Veces** | **Siempre** |
| 1 | He tenido dos o más parejas sexuales en un mismo periodo de tiempo. |  |  |  |  |
| 2 | He tenido relaciones sexuales con más de una persona al mismo tiempo (ej. Tríos, cuartetos, orgías, etc.). |  |  |  |  |
| 3 | He tenido sexo con más de una persona en un mismo día. |  |  |  |  |
| 4 | Cuando he estado en relaciones estables (ej. Pololo/a, novio/a, esposo/a, etc.), he tenido sexo con otras personas. |  |  |  |  |
| 5 | Si contara las parejas sexuales que he tenido, no me alcanzarían con los dedos de la mano. |  |  |  |  |
| 6 | Creo que he tenido sexo con más personas que el común de la gente. |  |  |  |  |
| 7 | He tenido sexo con varias(os) amigas (os) “con ventaja”. |  |  |  |  |
| 8 | He tenido encuentros sexuales esporádicos o “touch and go”. |  |  |  |  |
| 9 | He recibido semen y/o fluido vaginal en mi boca. |  |  |  |  |
| 10 | He sangrado y/o he tenido contacto con sangre de otra persona durante el sexo. |  |  |  |  |
| 11 | He tenido algún tipo de penetración antes de usar preservativo. |  |  |  |  |
| 12 | He tenido contacto entre genitales (roces) antes del uso del preservativo. |  |  |  |  |
| 13 | He tenido sexo con preservativo sin chequear si está en buen estado y/o su fecha de vencimiento. |  |  |  |  |
| 14 | He tenido sexo con preservativo sin asegurarme de que esté bien puesto. |  |  |  |  |
| 15 | Durante una pérdida de erección momentánea, hemos vuelto a tener penetración sin cambiar de preservativo. |  |  |  |  |
| 16 | He iniciado un encuentro sexual con preservativo, pero nos lo hemos quitado y continuado. |  |  |  |  |
| 17 | He tenido sexo con preservativo y éste se ha roto en el acto sexual. |  |  |  |  |
| 18 | He consumido alcohol y/o drogas para facilitar el sexo. |  |  |  |  |
| 19 | He tenido encuentros sexuales que no recuerdo producto del alcohol y/o drogas. |  |  |  |  |
| 20 | He accedido a tener relaciones sexuales con personas cuando estoy bajos los efectos del alcohol y/o drogas con las cuales no accedería normalmente. |  |  |  |  |
| 21 | Cuando he consumido alcohol y/o drogas en exceso he terminado teniendo relaciones sexuales. |  |  |  |  |

| **Indique con una X, por favor, con qué frecuencia usted se siente representado por las siguientes afirmaciones:** | | | | | |
| --- | --- | --- | --- | --- | --- |
| **Nº** | **ÍTEMS** | **Totalmente en Desacuerdo** | **En Desacuerdo** | **De Acuerdo** | **Totalmente De Acuerdo** |
| 1 | Me siento bien conmigo mismo(a) durante mis encuentros sexuales. |  |  |  |  |
| 2 | No cambiaría nada de mi vida sexual actual. |  |  |  |  |
| 3 | Me siento bien con la forma en que manejo mis deseos y fantasías sexuales. |  |  |  |  |
| 4 | Estoy bien conmigo mismo(a) en el ámbito sexual. |  |  |  |  |
| 5 | Estoy muy satisfecho(a) con mi vida sexual. |  |  |  |  |
| 6 | Me siento a gusto con mi cuerpo durante el sexo. |  |  |  |  |
| 7 | Me siento cómoda(o) durante mis encuentros sexuales. |  |  |  |  |
| 8 | Me siento feliz con mis actividades sexuales. |  |  |  |  |
| 9 | Me siento físicamente seguro(a) al momento de tener relaciones sexuales. |  |  |  |  |
| 10 | Me siento mentalmente seguro(a) al momento de tener relaciones sexuales. |  |  |  |  |
| 11 | Comienzo la intimidad con mi compañero(a) sexual solo cuando quiero. |  |  |  |  |
| 12 | Comunico a mi compañero(a) sexual cuando y como deseo ser estimulada(o). |  |  |  |  |
| 13 | Le expreso a mi(s) pareja(s) sexual cuando deseo que me acaricie(n). |  |  |  |  |
| 14 | Cuando algo me disgusta en el acto sexual se lo comunicó a mi(s) pareja(s). |  |  |  |  |
| 15 | Me mantengo firme a las presiones de mi(s) pareja(s) si no quiero tener relaciones sexuales o algún tipo de intimidad. |  |  |  |  |
| 16 | Solo realizo prácticas sexuales que deseo. |  |  |  |  |
| 17 | Tengo sexo solamente cuando quiero, incluso si mi(s) pareja(s) insiste(n) en tenerlo. |  |  |  |  |
| 18 | Manifiesto cuáles son mis sentimientos, afectos y deseos sexuales. |  |  |  |  |
| 19 | Soy clara(o) con mi(s) pareja(s) en relación a los aspectos sexuales de mi vida. |  |  |  |  |
| 20 | Expreso mis deseos sexuales con comodidad. |  |  |  |  |
| 21 | Pido lo que quiero durante una relación sexual. |  |  |  |  |
| 22 | Elijo mis encuentros sexuales. |  |  |  |  |
| 23 | Me pongo de acuerdo con mi(s) pareja(s) en las posiciones que disfruto en el acto sexual. |  |  |  |  |
| 24 | Con mi(s) pareja(s) nos respetamos si no tenemos ganas de tener sexo. |  |  |  |  |
| 25 | Con mi(s) pareja(s) conversamos si nos disgusta algo de la relación sexual. |  |  |  |  |
| 26 | Le(s) digo a mi(s) pareja(s) donde quiero que me toque(n) cuando tenemos sexo. |  |  |  |  |
| 27 | Cuando tengo fantasías sexuales, se la(s) digo a mi(s) pareja(s) para realizarla(s). |  |  |  |  |
| 28 | Creo que sé estimular bien a mi(s) pareja(s) |  |  |  |  |
| 29 | Identifico lo que mi(s) pareja(s) desea(n) durante los encuentros sexuales. |  |  |  |  |
| 30 | Creo que mi cuerpo es sexualmente satisfactorio para mí(s) pareja(s) sexual(es). |  |  |  |  |
| 31 | Considero que mi cuerpo es sexualmente satisfactorio para mí. |  |  |  |  |
| 32 | Mis encuentros sexuales son satisfactorios para mí y mi(s) pareja(s) |  |  |  |  |
| 33 | Creo que tengo un buen número de cualidades en el ámbito sexual |  |  |  |  |
| 34 | Me siento seguro(a) cada vez que tengo que empezar una relación sexual. |  |  |  |  |
| 35 | Me desenvuelvo de buena forma en el ámbito sexual. |  |  |  |  |
| 36 | Creo en mis capacidades y habilidades sexuales. |  |  |  |  |
| 37 | Soy capaz de satisfacer mis fantasías y deseos sexuales. |  |  |  |  |
| 38 | Tengo las capacidades para obtener gratificación sexual. |  |  |  |  |
